# miR-21 silencing ameliorates experimental autoimmune encephalomyelitis by promoting the differentiation of IL-10-producing B cells

**DOI:** 10.18632/oncotarget.21578

**Published:** 2017-10-06

**Authors:** Hui Wang, Wenrong Xu, Qixiang Shao, Qing Ding

**Affiliations:** ^1^ Jiangsu Key Laboratory of Medical Science and Laboratory Medicine, Department of Immunology, School of Medicine, Jiangsu University, Zhenjiang, Jiangsu, P.R. China; ^2^ Key Laboratory of Laboratory Medicine of Jiangsu Province, School of Medicine, Jiangsu University, Zhenjiang, Jiangsu, P.R. China

**Keywords:** microRNA-21, interleukin-10, regulatory B cells, experimental autoimmune encephalomyelitis

## Abstract

IL-10-producing regulatory B (IL-10^+^ Breg) cells promote tolerance in autoimmune diseases and transplantation. However, it remains unclear whether microRNAs are involved in the development of IL-10^+^ Breg cells. Here, we found that microRNA-21 (miR-21) acts as an upstream regulator of IL-10 by targeting the 3' untranslated region of IL-10 mRNA. We also demonstrated that IL-10^+^ Breg cells exhibit lower miR-21 expression than non-Breg cells and that miR-21 acts as a potent negative regulator of the differentiation of IL-10^+^ Breg cells. Accordingly, specific inhibition of miR-21 using antisense oligonucleotides markedly promoted B cell IL-10 expression. Thus, IL-10 is a direct target of miR-21. Moreover, silencing of miR-21 significantly alleviated the severity of experimental autoimmune encephalomyelitis (EAE), and this change was associated with an increase in the number of IL-10^+^ Breg cells. Finally, we demonstrated that miR-21-silenced B cells exert their suppressive activity through effector T cells in an IL-10-dependent manner.

Thus, we characterized a B cell-intrinsic microRNA pathway that inhibits the differentiation of IL-10^+^ Breg cells and promotes autoimmunity. miR-21 silencing therefore represents a new therapeutic strategy for the treatment of autoimmune diseases.

## INTRODUCTION

MicroRNAs (miRNAs) are small (∼22-nucleotide) non-coding RNAs that regulate gene expression through translational repression and mRNA degradation [1]. miRNAs can specifically recognize their target mRNAs via a ‘seed region’, which consists of the 2^nd^ through 8th nucleotides of the miRNA.

MiRNAs play important roles in many biological processes, including hematopoietic development, immunity, and carcinogenesis. The abnormal expression of miRNAs can lead to various diseases, including cancer and autoimmune diseases [2]. The term ‘oncomiRs’ refers to a series of specific miRNAs that function as oncogenes or tumor suppressor genes. OncomiR miRNA-21 (miR-21) has been found to be significantly overexpressed in tumors, such as breast, lung, colon, gastric and pancreatic cancers [3]. Another target of miR-21 is PTEN, which is a known tumor suppressor [4]. Over-expression of miR-21 may induce a decrease in the levels of PDCD4, a well-known tumor suppressor molecule associated with poor outcomes in cancer patients [5]. Thus, miR-21 plays an important role in tumor development.

miR-21 displays diverse functions in the immune system, and it plays an important role in fine-tuning the balance between immunity and tolerance. More recently, however, emerging data have established an important role for miR-21 in autoimmune inflammation [6, 7]. miR-21 expression in T cells was found to be closely associated with systemic lupus erythematosus (SLE), multiple sclerosis (MS) and psoriasis, among other disorders. In addition, knockdown of the expression of miR-21 reduced splenomegaly in a mouse model of lupus [8]. Moreover, miR-21-knockout mice exhibited both a defect in the Th17 response and strong resistance to DSS-induced colitis [9] and EAE [10]. Knockdown of miR-21 *in vivo* also dramatically reduced EAE disease and Th17 cell responses [10]. However, the effect of miRNAs on B cell differentiation remains unknown.

B cells play important roles in controlling the immune response. In addition to producing antibodies, B cells shape immune responses through antigen (Ag) presentation and the production of and co-stimulation via cytokines [11–13]. In this regard, regulatory B (Breg) cells producing IL-10 inhibit autoimmunity and allograft rejection and promote tumor growth [11–16]. In EAE in particular, B cells play a crucial role in recovery via the expression of the major immunosuppressive cytokine IL-10 [17].

Although several miR-21 targets have been examined in T cells, the function of miR-21 in B cells is largely unknown. Here, we report that IL-10 is a target of miR-21 and that miR-21 expression is specifically alleviated in IL-10-producing Breg cells. Moreover, silencing of miR-21 dramatically alleviated the clinical signs of EAE, and this change was associated with an increased number of IL-10^+^ Breg cells. Thus, we characterize an unknown B cell-intrinsic miRNA pathway that influences the development of IL-10^+^ Breg cells and identify miR-21 as a potential therapeutic target for the amelioration of autoimmune inflammation.

Given the observed therapeutic effect of miR-21 silencing on EAE disease, knockdown of the expression of miR-21 *in vivo* may be an effective therapeutic approach to the treatment of autoimmune diseases.

## RESULTS

### IL-10-producing Breg cells exhibit lower miR-21 expression than non-Breg cells

Elevated levels of miR-21 have been found in T cells in multiple autoimmune diseases [9, 10, 18]. However, the role of miR-21 in B cell differentiation and function has not been investigated yet. We first examined miR-21 expression levels in CD19^+^ B and CD4^+^ T cells and the role of miR-21 during the course of EAE. EAE was induced in male B6 mice by active immunization with the myelin oligodendrocyte glycoprotein (MOG)_35–55_ peptide; the incidence of EAE was 100% among all mice. The animals reached their peak disease state by day 20 and then entered a phase of spontaneous remission (data not shown).

**Figure 1:**
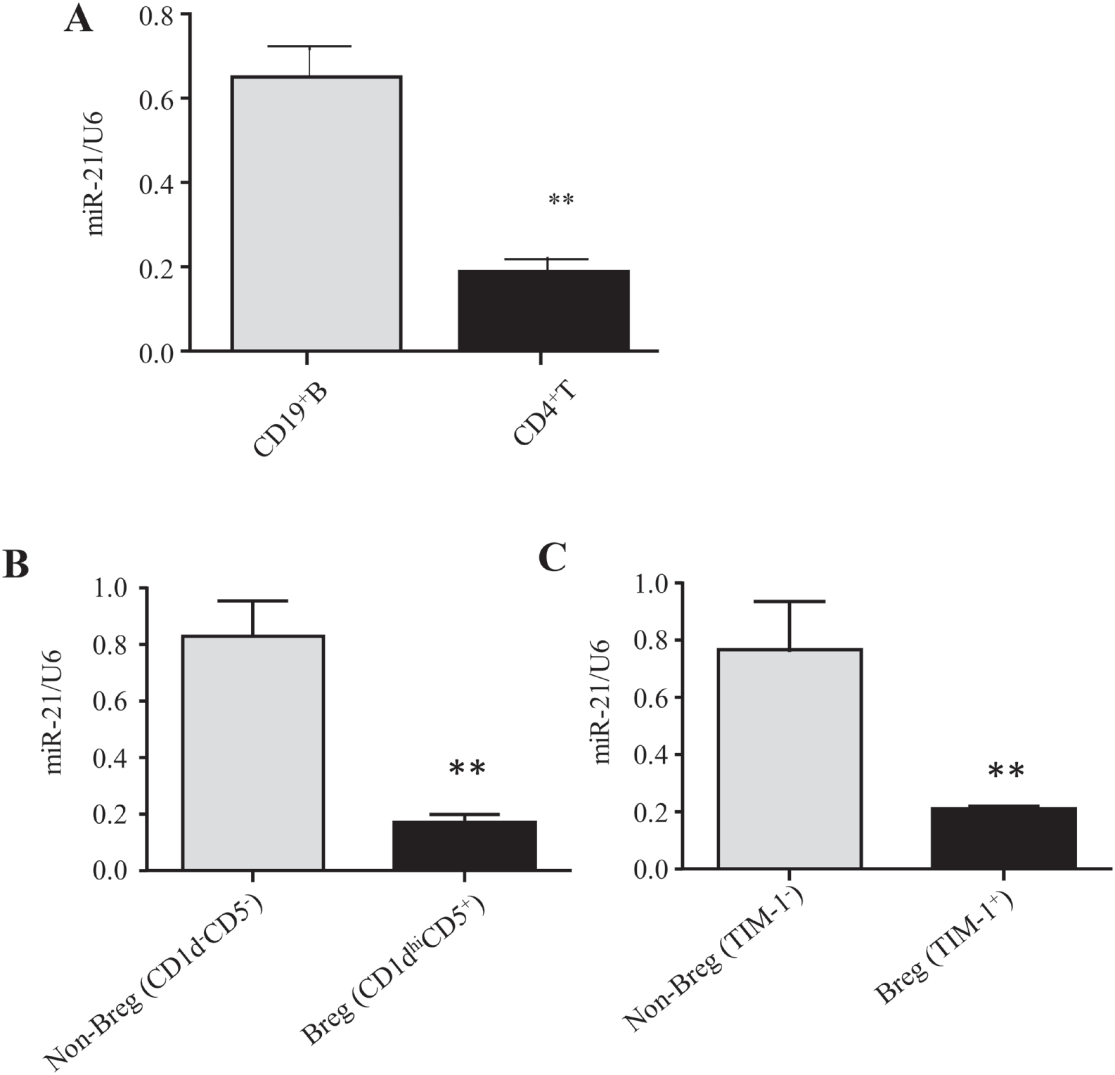
IL-10-producing Breg cells exhibit lower miR-21 expression than non-Breg cells **(A)** CD4^+^ T cells and CD19^+^ B cells were purified by MACS from the spleens of EAE mice at disease onset (day 14), and miR-21 expression was analyzed by qRT-PCR using U6 snRNA as an endogenous control. **(B, C)** CD5^+^CD1d^hi^ and CD5^-^CD1d^-^ B cells (B) and TIM-1^+^ and TIM-1^-^ B cells (C) were sort-purified from the spleens of EAE mice at the onset stage (day 14), and miR-21 expression was analyzed by qRT-PCR using U6 snRNA as an endogenous control. The data are representative of 3 independent experiments. The bars and error bars represent the mean ± SEM. ^**^P < 0.01.

For efficient analysis of mouse miR-21 expression, CD19^+^ B and CD4^+^ T cells were purified from the splenocytes of EAE mice at the disease-onset stage (day 14) by magnetic-activated cell sorting (MACS, StemCell) for quantitative reverse transcription (RT) PCR (qRT-PCR) analysis. Analysis of miR-21 revealed that its expression in spleen B cells from EAE mice was ∼4-fold higher than in CD4^+^ T cells (Figure 1A).

B cells mediate recovery from EAE via the expression of IL-10. IL-10-producing Breg cells, predominantly CD1d^hi^CD5^+^, are important for EAE disease recovery [19, 20]. Moreover, TIM-1 has been suggested to be an inclusive marker of IL-10-producing Breg (TIM-1^+^ Breg) cells [21].

To compare miR-21 expression levels between Breg and non-Breg cells, B10^+^ cells (CD1d^hi^CD5^+^CD19^+^) and B10^−^ cells (CD1d^−^CD5^−^CD19^+^) were purified from C57BL/6 mouse splenocytes by fluorescence-activated cell sorting (FACS) (Supplementary Figure 1) and used for subsequent qRT-PCR analysis. The results show that the levels of miR-21 expression were ∼5-fold lower in B10^+^ cells than in B10^-^ cells (Figure 1B). We also examined miR-21 expression in TIM-1^+^ Breg cells. TIM-1^+^ Breg (CD19^+^TIM-1^+^) and non-Breg (CD19^+^TIM-1^-^) cells were sort-purified from C57BL/6 mouse splenocytes and used for subsequent qRT-PCR analysis. The expression levels of miR-21 were ∼4-fold lower in TIM-1^+^ Breg cells than in non-Breg cells (Figure 1C), suggesting again that IL-10- producing Breg cells exhibit much lower miR-21 expression than IL-10^-^ B cells.

Based on these results, we hypothesized that miR-21 is negatively correlated with IL-10 expression and might participate in the differentiation of IL-10-producing Breg cells.

### miR-21 acts as a potent negative regulator of IL-10-producing B cells, and its inhibitor promotes B cell IL-10 expression

**Figure 2:**
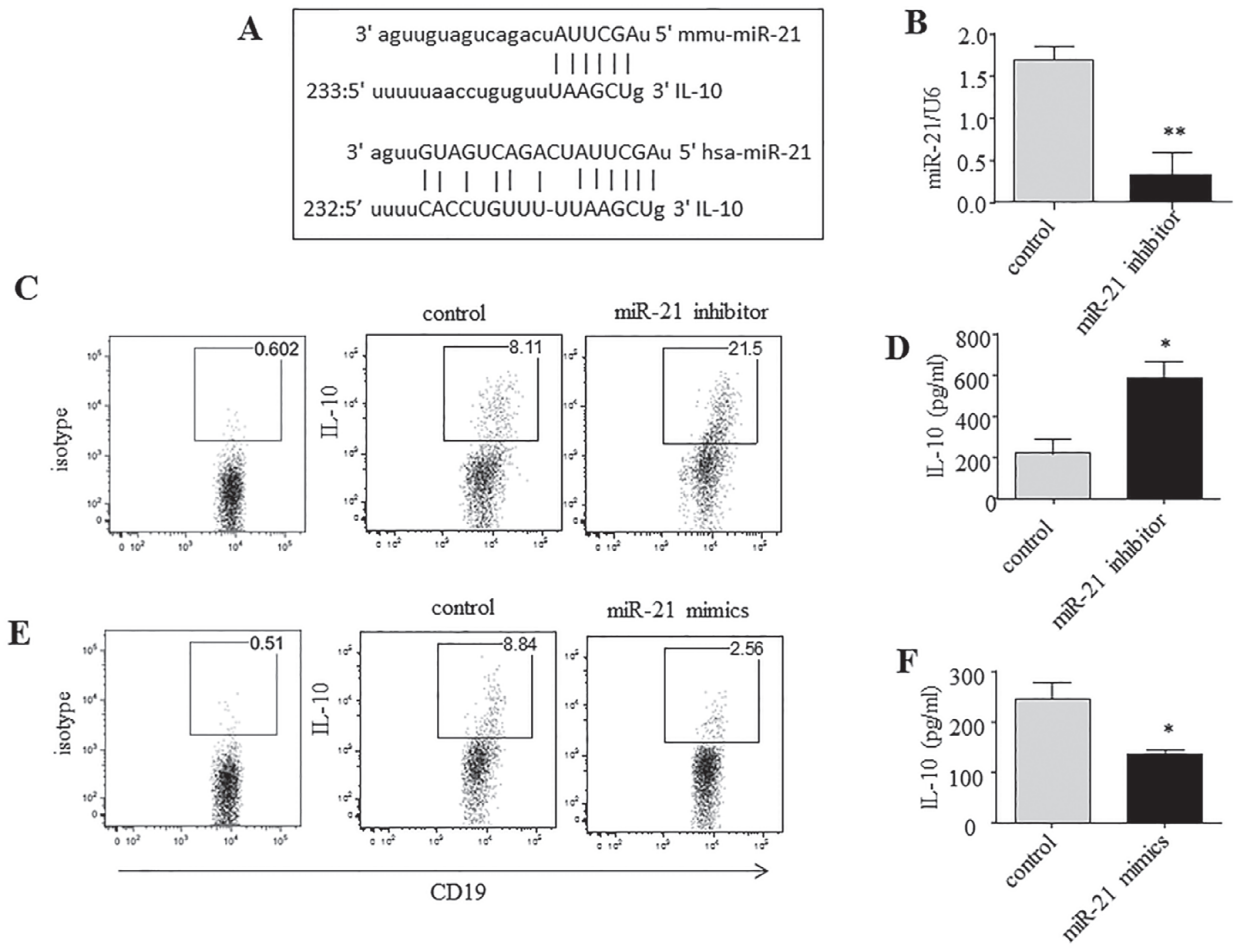
miR-21 acts as a potent negative regulator of IL-10-producing B cells **(A)** miR-21 is aligned with the highly conserved 3’UTR of IL-10 mRNA. **(B, C, E)** CD19^+^ B cells were purified by MACS from naive B6 mice, cultured for 24 h under LPS/PMA/ionomycin/monensin conditions, and transfected by nucleofection with miR-21 inhibitor, mimic or negative-control oligonucleotides. (B) miR-21 expression was analyzed by qRT-PCR using U6 snRNA as an endogenous control. (C, E) IL-10 levels were measured by intracellular staining and flow cytometric analysis. The numbers represent the frequencies of CD19^+^ B cells. **(D, F)** CD19^+^ B cells were cultured as described in B, C, and E, except monensin was not added. The culture supernatants were collected after 24 h and used for the estimation of IL-10 secretion by ELISA. The data are representative of 3 independent experiments. ^*^P < 0.05 and ^**^P < 0.01.

We then addressed whether B cell-intrinsic miR-21 was required for the differentiation of IL-10^+^ Breg cells. To determine if IL-10 is a target of miR-21 in IL-10^+^ Breg cell polarization, the miRNA target prediction program miRWalk [22] was applied to identify potential targets of miR-21. miR-21 was found to potentially act as an upstream regulator of IL-10 by targeting the 3'-untranslated region (3’-UTR) of IL-10 mRNA (Figure 2A).

Synthetic miRNA inhibitors are small single-stranded RNA molecules designed to inhibit specific miRNAs. These inhibitors are antisense oligonucleotides that bind to mature miRNAs and that have been used effectively to inhibit miRNA function. Here, the effect of miR-21 on IL-10 gene expression was examined using an inhibitor of miR-21. After transfection with a specific miR-21 inhibitor *in vitro* for 24 h, B cells exhibited a marked (∼80%) decrease in miR-21 expression (Figure 2B). Subsequently, the cells were subjected to intracellular staining and flow cytometric analysis, by which we observed a dramatic increase (∼2-fold) in IL-10 expression in miR-21-silenced B cells cultured under Breg-polarizing conditions (Figure 2C). To further confirm this effect, we collected the cell culture supernatants and assayed them for released IL-10 by enzyme-linked immunosorbent assay (ELISA). Markedly higher levels of IL-10 were observed in the supernatants of the miR-21 inhibitor-transfected B cells than in those of the negative-control-transfected B cells (Figure 2D). In contrast, transfection of an miR-21 mimic greatly decreased the levels of both intracellular IL-10 production and IL-10 secretion by B cells (Figure 2E, 2F). Taken together, these results suggest that miR-21 inhibits IL-10 and that its deletion results in increased IL-10 production by B cells. Thus, we identified a previously unknown miRNA pathway that affects the development of IL-10^+^ Breg cells.

### miR-21 silencing significantly ameliorates the clinical symptoms of EAE, and this change is associated with an increase in the number of IL-10^+^ Breg cells

We next examined whether systemic administration of an miR-21 inhibitor could protect mice against EAE. A highly specific and biologically stable miRNA-inhibitory antagomiR was used for the *in vivo* inhibition of miR-21 function. AntagomiRs are a class of chemically engineered antisense RNA oligonucleotides that prevent other molecules from binding to a specific miRNA target and that are used to silence endogenous miRNA. Usually, antagomiRs are modified in some way, such as via full-length nucleotide 2’-methoxy(2’-O-Me) modification or the addition of phosphorothioates (PS) at both the 5' and the 3' ends, to make them more resistant to degradation. In addition, antagomiRs have cholesterol tags (Chol) at their 3′ end to enable efficient, direct uptake of antagomiRs into viable cells via the cell membrane. Herein, “antagomiR-21” is used to refer to a small synthetic RNA complementary to the specific miRNA target miR-21.

In this study, mouse antagomiR-21 was used to silence miR-21 in EAE mice *in vivo*. We investigated whether silencing of miR-21 *in vivo* with antagomiR-21 could promote IL-10-producing Breg cells and ameliorate the clinical symptoms of EAE.

**Figure 3:**
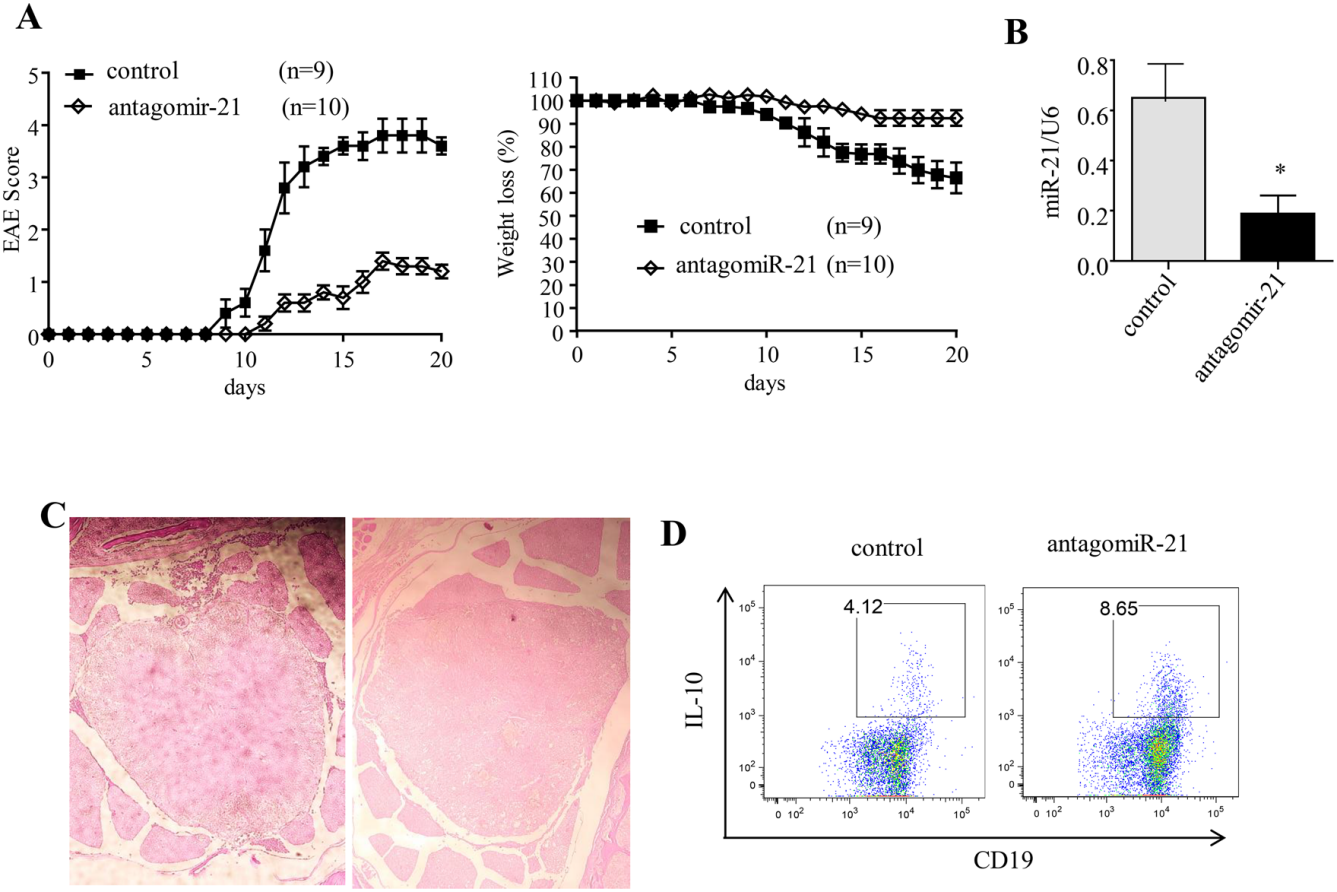
Silencing of miR-21 ameliorates the clinical severity of EAE **(A)** Mean clinical score and weight loss of B6 EAE mice treated with antagomiR-21 or the negative control. The data represent one experiment and are presented as the mean ± SEM. **(B)** The miR-21 expression level was analyzed by qRT-PCR using the U6 snRNA as an endogenous control. **(C)** Spinal cord histology was analyzed on day 14 after immunization. The degree of inflammation in the negative-control-treated mice (left) and antagomiR-21-treated mice (right) was determined by H&E staining. The pictures shown are representative of the spinal cord histology of 3 mice in each group. **(D)** Splenocytes obtained from antagomiR-21-treated and negative-control-treated EAE mice were restimulated with PMA/ionomycin/monensin/LPS for 5 h. The data are representative of 3 independent experiments. ^*^P < 0.05.

EAE was induced in B6 mice by subcutaneous immunization with an emulsion of the MOG_35–55_ peptide in complete Freund’s adjuvant (CFA). The mice that received negative control antagomiRs (non-targeting control antagomiR oligonucleotides) developed EAE. As in wildtype (wt) B6 mice, the incidence of EAE was ∼100%. These mice that received negative control antagomiRs displayed peak stages of disease by day 20 and then entered remission (Figure 3A). The systemic delivery of antagomiR-21 by four intravenous injections of 30 μg/mouse efficiently inhibited B-cell-expressed miR-21 (Figure 3B). The mice with antagomiR-21 treatment also developed a significantly milder clinical course of EAE than the mice that were treated with negative control antagomiRs. This finding demonstrated the therapeutic efficacy of antagomiR-21 in EAE.

When the spinal cord was fixed with 4% formaldehyde and stained with hematoxylin and eosin (H&E) to examine leukocyte infiltration during the course of EAE, infiltration was found to be significantly reduced in the antagomiR-21-treated mice (Figure 3C). This observed change correlated closely with severity of EAE disease.

In addition, we examined the effect of antagomiR-21 on B cell IL-10 expression *in vivo*. Spleen cells were isolated from MOG_35–55_ peptide-immunized mice treated with antagomiR-21 or the negative control antagomiRs and stimulated *in vitro* with LPS/ionomycin/PMA/monensin; IL-10 was then detected by an intracellular flow cytometric assay. We observed that B cells from antagomiR-21-treated mice had an approximately 2.5-fold increase in IL-10 expression over that of the control-treated mice, suggesting that IL-10^+^ Breg cells play a critical role in the alleviation of EAE induced by antagomiR-21 treatment (Figure 3D). These data raised the question of whether antagomiR-21-induced IL-10^+^ Breg cells could directly ameliorate EAE and suppress the production of pro-inflammatory cytokines.

### miR-21-silenced B cells exhibit suppressive activity *in vivo*

**Figure 4:**
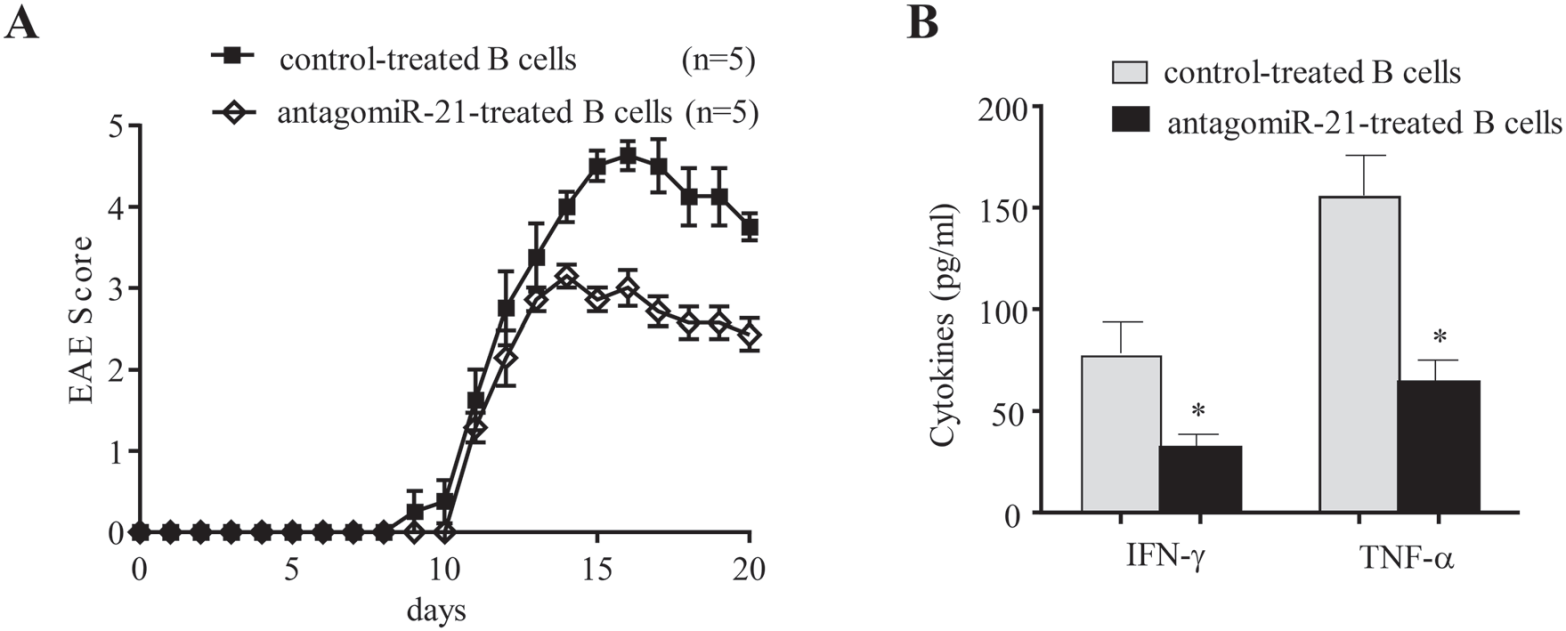
Transfer of miR-21-silenced B cells ameliorates the clinical severity of EAE and inhibits pro-inflammatory cytokine expression **(A)** CD19^+^ B cells were purified by MACS from antagomiR-21-treated or negative-control-treated B6 mice on day 14 after EAE immunization; 1×10^7^ purified B cells were then adoptively transferred into B6 EAE mice on days 1, 5 and 10 after immunization, and the EAE score was assessed daily. The mean clinical scores of the EAE mice are shown; the data represent one experiment and are presented as the mean ± SEM. **(B)** Serum was collected from mice as described in A on day 14 after EAE induction, and ELISA was performed to detect the expression levels of the cytokines IFN-γ and TNF-ɑ. The serum samples were collected from 3 mice per group, and the results are expressed as the mean ± SEM. ^*^P < 0.05.

Next, we set up an adoptive transfer model to address whether the amelioration of EAE in antagomiR-21-treated mice was mainly induced by B cells. CD19^+^ B cells were purified by MACS from antagomiR-21-treated or negative-control-treated wt B6 EAE mice on day 14 after immunization; 1×10^7^ purified B cells were then adoptively transferred into the B6 EAE mice on days 1, 5 and 10 after immunization. The EAE score was assessed daily, and serum levels of various pro-inflammatory cytokines, including IFN-γ and TNF-ɑ, were measured using ELISA. The results showed that adoptive transfer of antagomiR-21-treated B cells efficiently alleviated the clinical severity of EAE (Figure 4A) and that this change was associated with down-regulation of serum pro-inflammatory cytokines, including IFN-γ and TNF-ɑ (Figure 4B). Collectively, these data suggest that the B cells of antagomiR-21-treated mice achieve potent regulatory activity and efficiently alleviate EAE severity by inhibiting pro-inflammatory cytokines. These data raised the question of whether antagomiR-21-treated B cells ameliorate EAE and suppress the expression of pro-inflammatory factors by producing IL-10.

### miR-21-silenced B cells exert their regulatory activity in an IL-10-dependent manner

To further evaluate the regulatory mechanisms of antagomiR-21 treatment and the role of B-cell-expressed IL-10, we set up a co-culture system to study the effects of B cells from antagomiR-21-treated EAE mice on effector T (Teff) cell responses [23]. First, MACS-purified splenic B cells from antagomiR-21-treated EAE mice were activated with anti-CD40 and LPS, and second, the cells were added to cultures containing both CFSE-labeled CD4^+^ T cells from EAE mice and the MOG_35–55_ peptide. After 72 h of culture, T cell proliferation, as examined based on CFSE dilution, was analyzed by flow cytometry.

**Figure 5:**
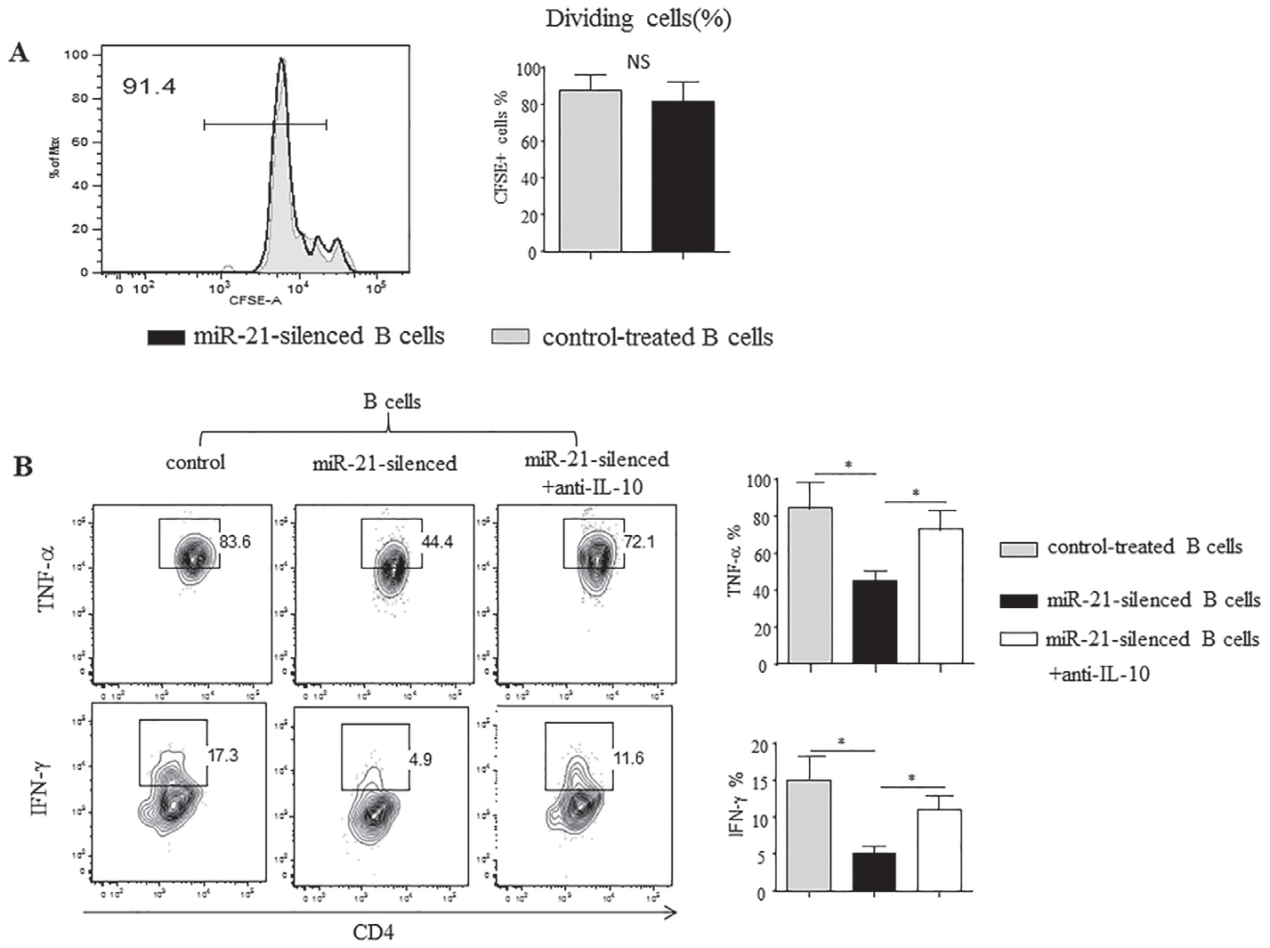
Silencing of miR-21 in B cells alters T cell cytokine profiles Purified splenic CD19^+^ B cells from control- or antagomiR-21-treated mice with EAE (day 14) were stimulated with agonistic CD40 mAb for 48 h, with LPS added during the final 5 h. Purified CD4^+^ T cells from wt B6 mice with EAE were labeled with CFSE and cultured with CD40/LPS-stimulated B cells in the presence of the MOG_35–55_ peptide for 72 h. **(A)** The cultured cells were stained for CD4 and analyzed for CFSE dilution by flow cytometry. Representative frequencies of dividing CFSE-labeled cells are shown. The bar graphs indicate the mean frequencies of dividing CFSE-labeled CD4^+^ T cells (± SEM) from 3 mice. **(B)** PMA, ionomycin, and BFA were added during the final 5 h of culture. Cytokine production by CD4^+^ T cells was determined by intracellular staining and flow cytometric analysis. The numbers indicate the percentage of T cells within the indicated gates among all CD4^+^ T cells in the sample. The bar graphs indicate the mean (± SEM) percentage of cytokine-producing CD4^+^ T cells from 3 mice. ^*^P < 0.05.

CD4^+^ T cells co-cultured with CD19^+^ B cells from either antagomiR-21-treated or negative-control-treated mice displayed similar proliferation in the presence of the MOG_35–55_ peptide (Figure 5A). Thus, antagomiR-21-treated B cells did not inhibit T cell proliferation. However, CD4^+^ T cells cultured with antagomiR-21-treated B cells expressed markedly decreased expression of IFN-γ and TNF-ɑ compared with CD4^+^ T cells cultured with control-treated B cells (Figure 5B). These effects were dependent on B cell IL-10 expression because an anti-IL-10 neutralizing antibody efficiently reversed the effects on IFN-γ and TNF-ɑ production by CD4^+^ T cells. Thus, antagomiR-21-treated B cells do not suppress T cell proliferation, but rather inhibit Teff cell cytokine expression, and this suppressive activity is dependent on IL-10.

## DISCUSSION

Recently, miRNAs have emerged as key regulators of immune responses. miRNAs are highly pleiotropic, and a given miRNA can target different mRNAs to have a greater influence on the cell. In addition, multiple individual miRNAs can converge upon the same mRNA target [2, 24, 25]. In the case of miR-21, a number of targets have been experimentally validated, and most of them are tumor suppressors; notable targets include PDCD4, FasL, and PTEN [4, 5, 7, 26, 27]. Here, for the first time, we identified IL-10 as a target of miR-21 and established that miR-21 is a signal that negatively regulates Breg cell development.

**Figure 6:**
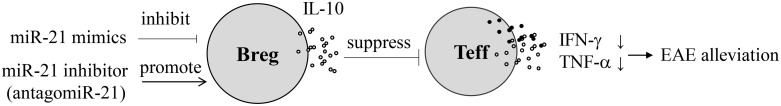
Silencing of miRNA-21 ameliorates EAE 1) miR-21 acts as a potent negative regulator of IL-10-producing B cells, and miR-21 inhibition promotes B cell IL-10 expression. 2) Silencing of miR-21 significantly ameliorates the clinical severity of EAE, and this change is associated with an increase in the number of IL-10^+^ Breg cells. 3) miR-21-silenced B cells exhibit suppressive activity *in vivo* and exert their regulatory activity in an IL-10-dependent manner.

In this research, we demonstrated that in EAE, the major function of miR-21 is to inhibit IL-10^+^ Breg cells. Targeting miR-21 resulted in efficient induction of the generation of IL-10^+^ Breg cells, which in turn alleviated EAE by suppressing Teff cells (Figure 6). To our knowledge, this is the first report describing the role of miR-21 in regulating Breg cell and Teff cell responses. Previous studies on miR-21 have primarily focused on its function in Teff cells and tumorigenesis [18, 28–30]; the finding that miR-21 dominantly inhibits Breg cells is thus unanticipated. A previous study concerning the regulation of Breg cell differentiation mainly focused on regulation by cytokines and co-stimulators [31–33]; therefore, our finding that this important step in immune regulation is modulated by a unique miRNA has generated a novel paradigm for immunotherapy.

miR-21 is highly up-regulated in various autoimmune and allergic diseases [7, 34]. In the present study, based on predictive algorithms as well as *in vitro* and *in vivo* studies, we proposed that miR-21 targets IL-10. Moreover, we evaluated the activity of miR-21 in the context of adaptive immunity by examining the severity of EAE in miR-21-knockdown mice. Interestingly, miR-21-knockdown mice had less severe EAE, which was associated with an increased number of IL-10^+^ Breg cells. Thus, miR-21-induced attenuation of IL-10 signaling has potent regulatory effects on adaptive immunity. These findings indicate a crucial role for miRNA-mediated regulation of the differentiation of IL-10^+^ Breg cells.

The IL-10-knockout mouse model of inflammatory bowel disease (IBD) is a useful tool to analyze the mechanisms underlying IBD [35]. It has been demonstrated that the development of IBD in IL-10-knockout mice is accompanied by up-regulated expression of miR-21 [9, 36]. The specific up-regulation of miR-21 in IL-10-knockout mice and the negative correlation between miR-21 and IL-10 expression support our conclusion that IL-10 is a target of miR-21.

In addition to its role in regulating IL-10 expression, miR-21 may modulate other signaling pathways. For example, there is abundant evidence that miR-21 regulates apoptotic signaling pathways by targeting genes such as PDCD4 and PTEN, which have also been implicated in immunity.

miR-21 has been shown to be closely associated with autoimmune diseases. Given that the loss of Breg cells exacerbates disease symptoms in models of EAE [31, 37, 38], the capability of miR-21 to suppress Breg cells may be an additional mechanism underlying its action against autoimmune disorders. In agreement with the theory that miRNA activity is controlled by the particular cellular context, we highlight a new role for miR-21, which suppresses signaling through this pathway [39]. Our findings specifically demonstrate that miR-21 augments adaptive immunity.

Murugaiyan et al.[10] reported that miR-21 mediates EAE development by promoting the Th17 response and that IL-17 production in miR-21-deficient and miR-21-silenced T cells was significantly reduced. Moreover, the authors claimed that Smad-7 is a direct target of miR-21 and that miR-21 augments Th17 responses by inhibiting Smad-7. Here, we report that IL-10 is a direct target of miR-21, which mediates EAE by inhibiting IL-10^+^ Breg cells. It is well known that IL-10 suppresses Th17 responses [40, 41]. B cell-produced IL-10 is important for the suppression of Th17 differentiation and for recovery from autoimmune inflammation [42]. Our current results, along with previous work, support the induction of IL-10-producing Breg cells by miR-21 silencing, which alleviates EAE via inhibition of the Th17 response.

Collectively, our data indicated that miR-21 was a regulator of Breg cell differentiation in a model of autoimmune disease. Despite the multistage nature of immune regulation, Breg cell differentiation appears to be dependent on miR-21. Our findings have profound implications for diseases associated with defective Breg cell numbers or function. Thus, silencing of miR-21 and its effect on the Breg/Teff cell pathway may lead to potentially influential therapies and preventative strategies. Taken together, our findings afford novel mechanistic insights into Breg cell differentiation, Breg/Teff cell interaction and the development of autoimmune diseases.

## MATERIALS AND METHODS

### Induction and evaluation of EAE

Male C57BL/6 mice (6-8 weeks old), originally obtained from the Jackson Laboratory, were injected s.c. in both flanks with 100 μg of the MOG_35–55_ peptide (MEVGWYRSPFSRVVHLYRNGK) that was dissolved in PBS, emulsified in an equal volume of CFA (Difco), and supplemented with 5 mg/ml heat-killed mycobacterium tuberculosis H37Ra (Difco). The mice were also injected twice i.p. with 200 ng of pertussis toxin (List Biological Laboratories), once on the day of immunization and once 24 h later. Clinical assessment of EAE was performed daily after disease induction according to the following criteria: 0, no disease; 1, tail paralysis; 2, hind limb weakness or partial paralysis; 3, complete hind limb paralysis; 4, forelimb and hind limb paralysis; and 5, moribund state. Disease scores over the course of the 30-day experiment were totaled for each animal, and the mean for the experimental group was expressed as a cumulative EAE score.

### RNA isolation, cDNA synthesis, and real-time RT-PCR analysis

Total RNA was extracted using an RNeasy Micro Kit (Qiagen) following the manufacturer’s protocol. For analysis of miR-21-5p expression, U6 was used as an endogenous control. The RT primers for U6 and miR-21a-5p are listed in Table 1. SuperScript^™^ III Reverse Transcriptase (Invitrogen) was used to generate cDNA. miR-21a-5p transcripts were quantified by real-time PCR analysis using SYBR Green as the detection agent. PCR was performed with the ViiA 7 Real-Time PCR System (Applied Biosystems). All components of the PCR mix were purchased from Arraystar and used according to the manufacturer’s instructions. The thermal cycler conditions consisted of one cycle of denaturation at 95°C for 10 min followed by 40 cycles of 95°C for 10 s and 60°C for 1 min. The specificity of the RT-PCR was ensured by the generation of melting curves. The relative expression of miR-21 was calculated using the Ct method and normalized to uniformly expressed U6 snRNA. For all reactions, each condition was tested in triplicate.

**Table 1:** Sequences of miRNA-21 mimic, inhibitor, and RT-PCR primers

Reverse transcription		
U6 RT primer	5’-CGCTTCACGAATTTGCGTGTCAT-3’	
miR-21 RT primer	5’-GTCGTATCCAGTGCGTGTCGTGGAGTCGGCAATTGCACTGGATACGACTCAACA-3’	
qRT-PCR		
	sense primer	anti-sense primer
U6 primers	5’-GCTTCGGCAGCACATATACTAAAAT-3’	5’-CGCTTCACGAATTTGCGTGTCAT-3’
miR-21a-5p primers	5’-GGGGGGTAGCTTATCAGACTG-3’	5’-CAGTGCGTGTCGTGGAGT-3’
Mimic, inhibitor, and negative control		
miR-21a-5p mimic	5’-UAGCUUAUCAGACUGAUGUUGA-3’	
miR-21a-5p inhibitor	5’-UCAACAUCAGUCUGAUAAGCUA-3’ (antagomiR-21 with 3’-chol, 2’-ome, PS linkage)	
miR inhibitor NC	5’-CAGUACUUUUGUGUAGUACAA-3’ (3’-chol, 2’-ome, PS linkage)	

### Transfection and flow cytometry for intracellular cytokines

The miR-21 mimic, the miR-21 inhibitor and their respective control oligonucleotides were designed and chemically synthesized by GenePharma; the sequences are listed in Table 1. Purified B cells were seeded in 12-well plates at 5×10^5^ cells per well. The miR-21 mimic, inhibitor or corresponding negative control (50 nM) was transfected into cells for 24 h using RNAmate (GenePharma) in complete RPMI 1640 medium containing LPS (10 μg/ml), monensin (2 μm) and 10% FBS. The cells were then harvested and surface stained. Subsequent intracellular staining and flow cytometry for intracellular IL-10 expression were performed according to the manufacturer’s protocol (Pharmingen).

### AntagomiR-21 treatment

To silence miR-21 *in vivo*, antagomiR-21 or negative control (30 μg/mouse, GenePharma) was administered i.v. to mice on days 1, 5, 9, 13, and 17 after MOG_35–55_ peptide immunization [10]. The oligonucleotide sequences for the antagomiR-21 and the miRNA inhibitor negative control are listed in Table 1.

### *In vitro* T cell and B cell co-culture assays

MACS-purified splenic B cells (1×10^6^/ml) from antagomiR-21-treated or negative-control-treated EAE mice (day 14) were stimulated with agonistic anti-CD40 mAb (10 μg/ml, 48 h) and LPS (10 μg/ml, last 5 h of culture). MACS-purified CD4^+^ T cells (2×10^5^/ml) from wt EAE mice (day 14) were labeled with CFSE and cultured alone or with anti-CD40/LPS-stimulated B cells (1×10^6^/ml) in the presence of the MOG_35–55_ peptide (25 μg/ml) for 72 h [23]. After the cells were harvested, CFSE dilution was assessed as a measure of proliferation.

For the flow cytometric analysis of IFN-γ and TNF-ɑ, PMA (50 ng/ml), ionomycin (500 ng/ml), and brefeldin A (BFA, 5 μg/ml) were added during the final 4 h of culture. Cytokine production by CD4^+^ T cells was determined by intracellular cytokine staining and flow cytometry following the manufacturer’s instructions (Pharmingen).

### Determination of cytokine production

For cytokine analysis, cell cultures were prepared as described above, and the supernatants were collected at the indicated times and stored at -20°C. The supernatant concentrations of IFN-γ and TNF-ɑ were determined using cytokine ELISA kits (eBioscience). The results were analyzed spectrophotometrically at 450 nm using an ELISA microplate reader.

### Histology

For histopathological studies, spinal cords were dissected from mice, fixed with 10% formalin in PBS and embedded in a single paraffin block. Sections (6- to 10-μm-thick) were stained with H&E. Stained sections were evaluated for immune cell infiltration and demyelination.

### Statistics

Statistical analysis was performed using an unpaired 2-tailed Student’s *t*-test. A *P* value of less than 0.05 was considered statistically significant. Data are presented as the mean ± SEM. For EAE, groups were compared using linear regression analysis.

### Study approval

The animals were maintained under specific pathogen-free conditions in the animal facility of Jiangsu University School of Medicine. All mice were 6-8 weeks of age at the beginning of the experiments. All experiments were reviewed and approved by the Institutional Animal Care and Use Committee of Jiangsu University School of Medicine.

## SUPPLEMENTARY MATERIALS FIGURE


