# Supplementary material for: miR-21 silencing ameliorates experimental autoimmune encephalomyelitis by promoting the differentiation of IL-10-producing B cells
Source: Oncotarget. 2017 Oct 6;8(55):94069–79. doi: 10.18632/oncotarget.21578 (PMC5706856; doi:10.18632/oncotarget.21578)
Supplement: Supplementary file 1 [file oncotarget-08-94069-s001.pdf]

miR-21 silencing ameliorates experimental autoimmune encephalomyelitis by promoting the differentiation of IL-10-producing B cells

SUPPLEMENTARY MATERIALS

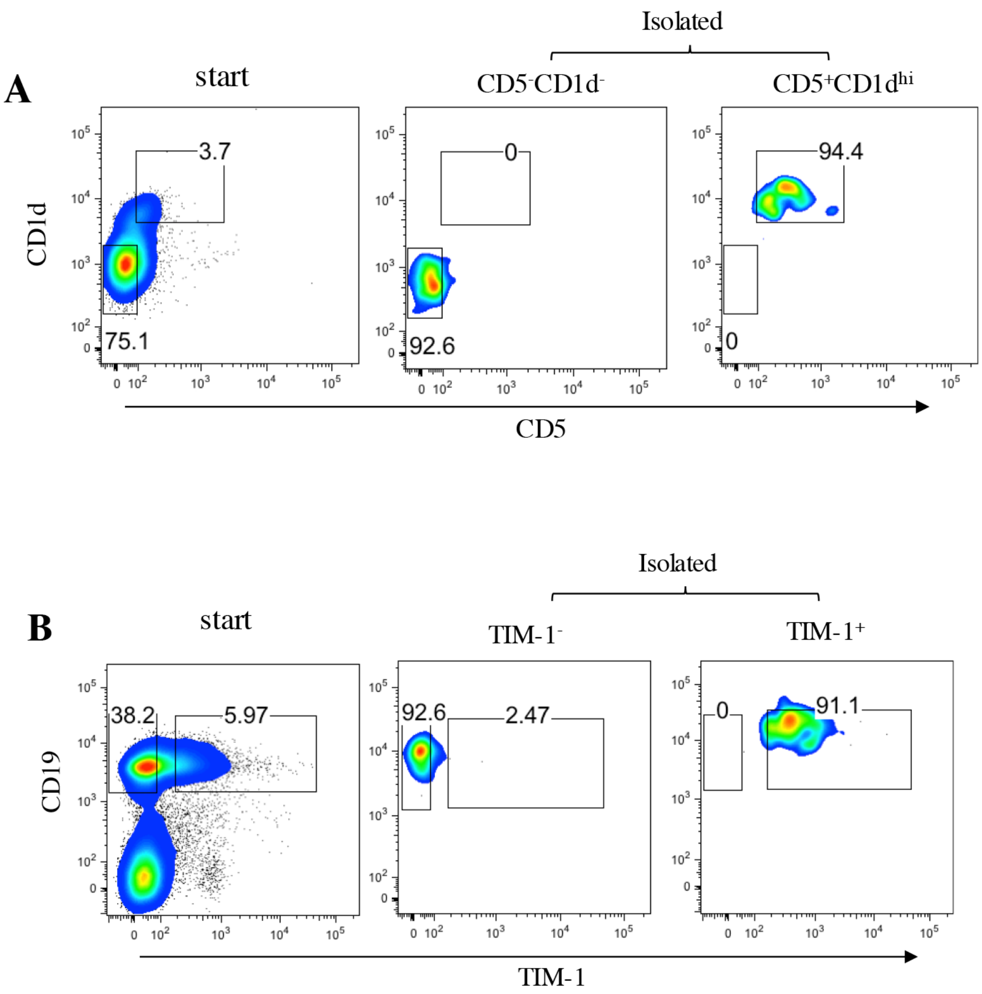

Supplementary Figure 1: (A) Purification of CD5$^+$CD1d^{hi} and CD5$^-$CD1d$^-$ CD19$^+$ B cells. Spleen cells, harvested from B6 mice at the onset stage of EAE (day 14 after immunization), were stained with anti- CD19/anti-CD5/anti-CD1d, and sort-purified into CD5$^+$CD1d^{hi}, CD5$^-$CD1d$^-$ B cells. The purity of isolated cell subsets was checked by flow cytometry. **(B)** Sort-Purification of TIM-1$^+$ and TIM-1$^-$ B cells. Spleens cells, harvested from B6 mice at the onset stage of EAE (day 14 after immunization), were stained with anti-CD19/anti-TIM-1, and sort-purified into TIM-1$^+$ and TIM-1$^-$ B cells. The purity of cell subsets was checked by flow cytometry.
